# Schmerztherapie in der deutschen spezialisierten ambulanten Palliativversorgung: Eine Querschnittsstudie zur Darstellung der aktuellen schmerzmedizinischen Versorgung von palliativen Patienten im häuslichen Umfeld

**DOI:** 10.1007/s00482-023-00693-x

**Published:** 2023-02-08

**Authors:** Christian Volberg, Julien Corzilius, Julian Maul, Astrid Morin, Martin Gschnell

**Affiliations:** 1 Klinik für Anästhesie und Intensivtherapie, Universitätsklinikum Marburg, Philipps-Universität Marburg, Marburg, Deutschland; 2 AG Ethik in der Medizin, Fachbereich 20, Dekanat Humanmedizin, Philipps-Universität Marburg, Marburg, Deutschland; 3 Klinik für Dermatologie und Allergologie, Hauttumorzentrum, Universitätsklinikum Marburg, Philipps-Universität Marburg, Marburg, Deutschland

**Keywords:** Palliativmedizin, SAPV, Schmerzmedikamente, Versorgungsforschung, Palliative Sedierung, Palliative medicine, SAPV, Pain medication, Health services research, Palliative sedation

## Abstract

**Hintergrund:**

Mithilfe der spezialisierten ambulanten Palliativversorgung (SAPV) können in Deutschland schwersterkrankte und sterbende Patienten im häuslichen Umfeld bis zum Lebensende adäquat versorgt werden. Es gibt für die Schmerztherapie jedoch keine einheitlichen Standards oder Vorgaben, wie diese am besten durchgeführt werden sollte.

**Ziel der Arbeit:**

Diese Arbeit dient der Grundlagenforschung im Bereich der Versorgungsforschung. Es soll dargestellt werden, welche Möglichkeiten (Einsatz verschiedener Berufsgruppen, Medikamente, alternativmedizinische Behandlungen etc.) die einzelnen SAPV-Teams für die Schmerztherapie nutzen, um daraus ableiten zu können, welche Strukturen als besonders effektiv angesehen werden können.

**Material und Methodik:**

Diese Querschnittsstudie wurde im Mai 2021 durchgeführt. Alle auf der Homepage der Deutschen Gesellschaft für Palliativmedizin (DGP) gelisteten SAPV-Teams (*n* = 307) wurden postalisch angeschrieben und um Teilnahme gebeten. Insgesamt antworteten 175 (57 %) Teams auf die Anfrage und konnten in die Auswertung eingeschlossen werden. Es wurde eine rein deskriptive Datenauswertung durchgeführt.

**Ergebnisse:**

Die Schmerztherapie in der deutschen ambulanten Versorgung palliativer Patienten basiert auf unterschiedlichen Bausteinen. Alle gängigen Schmerzmedikamente werden eingesetzt, vor allem aber Metamizol (99,4 %) als Nichtopioidanalgetikum, Morphin (98,3 %) aus der Reihe der Opiate und Pregabalin (96,6 %) als Koanalgetikum. Bei nichtbeherrschbaren Schmerzen führen 22,5 % der SAPV-Teams regelhaft eine palliative Sedierung zur Symptomlinderung durch.

**Diskussion:**

Diese Erhebung gibt als erste dieser Art einen generellen Überblick über die eingesetzten Verfahren zur Schmerztherapie in der ambulanten palliativmedizinischen Versorgung. Im Vergleich mit internationalen Studien stellt sich die Frage, ob eventuell einheitliche Therapieschemata und eine Reduktion der zur Verfügung stehenden Medikamente in den einzelnen SAPV-Teams zu einer Verbesserung der Patientenversorgung führen könnten.

## Abstract (en)

**Background:**

With the help of specialized outpatient palliative care teams (German abbreviation: SAPV), seriously ill and dying patients in Germany can be adequately cared for in their home environment until the end of their lives; however, there are no uniform standards or guidelines for well-executed pain management right now.

**Objective:**

This approach serves as basic research in the field of public health research. This is intended to present which methods (use of different professional groups, use of pain medications, alternative medical treatment etc.) the individual SAPV teams use for pain management. From this it can be deduced which procedures can be considered particularly effective.

**Material and methods:**

This cross-sectional study was conducted in May 2021. All German SAPV teams (*n* = 307) listed on the homepage of the German Association for Palliative Medicine (DGP) were contacted by post and invited to participate. A total of 175 teams (57%) responded to the request and were included in the evaluation. A descriptive data analysis was performed.

**Results:**

Pain management in the German outpatient care of palliative patients is based on several components. All common pain medications are used, but primarily metamizole (99.4%) as a non-opioid analgesic, morphine (98.3%) from the opiate series and pregabalin (96.6%) as a co-analgesic are mainly prescribed. If pain therapy fails, 22.5% of the SAPV teams perform palliative sedation for symptom control on a regular basis.

**Conclusion:**

This cross-sectional study is the first of its kind to provide a general overview of the treatment options for pain management in German outpatient palliative care. In comparison with international studies, the question arises as to whether uniform therapy schemes and a reduction in the medication available in the individual SAPV teams could lead to an improvement in patient care.

## Einleitung

Mit dieser Erhebung soll die Schmerztherapie in der ambulanten Versorgung palliativer Patienten durch die spezialisierte ambulante Palliativversorgung (SAPV) in Deutschland näher beleuchtet werden. Schmerzen treten bei an Krebs erkrankten Patienten besonders häufig mit einer Prävalenz von 70 bis 80 % auf. Dabei ist die Therapie leider oft nicht suffizient und Patienten werden nicht ausreichend behandelt [20]. Schmerz als Warn- und Schutzfunktion verliert bei Tumorerkrankungen diesen Stellenwert und belastet die betroffenen Patienten als unangenehmes Sinneserleben und schränkt die Lebensqualität unmittelbar ein [2]. Dabei ist das Schmerzempfinden individuell und kann nicht von außen ermessen und allenfalls bedingt objektiv gemessen werden. Trotzdem ist es wichtig, die Schmerzintensität bei der Aufnahme und im Verlauf der Behandlung mithilfe eines skalierten Messinstruments zu erfassen [20]. Für das Vorhandensein von Schmerzen muss kein anatomisches oder physiologisches Korrelat existieren. Schmerz kann in verschiedene Unterformen je nach Ursache und beteiligtem Nervensystem eingeteilt werden:

- Nozizeptiver Schmerz (durch Irritation von Schmerzrezeptoren)
  - Somatischer Schmerz (z. B. Haut, Muskeln, Knochen)
  - Viszeraler Schmerz (z. B. innere Organe)
- Neuropathischer Schmerz (durch Irritation peripherer oder zentraler Nerven)
- „Mixed pain“ (Mischform aus nozizeptivem und neuropathischem Schmerz) [14]

Abgesehen von dieser medizinischen Einteilung kann die Schmerzentstehung und -unterhaltung auch durch das bio-psycho-sozial-spirituelle Modell beschrieben werden. Hierbei spielen biologische, psychologische, soziale, kulturelle, spirituelle und funktionelle Ursachen mit in die Entstehung und Verarbeitung von Schmerz hinein. Durch diese erweiterte Herangehensweise und Betrachtung des Phänomens Schmerz wurde von Cicely Saunders der in der Palliativmedizin geläufige Begriff des „total pain“ geprägt. Diese Ausdrucksform beschreibt ein Nichtaushalten der Gesamtsituation, welches sich in Schmerz niederschlägt und die Person aktiv in ihrer biopsychosozialen Integrität bedroht [36]. Hierdurch wird erkenntlich, dass Schmerz nicht nur über die klassische medikamentöse Therapie behandelt werden sollte, sondern ein mehrdimensionales Behandlungskonzept notwendig ist. Dieses wird in der Palliativmedizin und Hospizarbeit z. B. durch die Zuhilfenahme von Psycho‑, Physio‑, Musik- und Kunsttherapie umgesetzt [14]. Bei Versagen jedweder Therapieform und weiter existierendem erheblichem Leidensdruck für den Patienten besteht in letzter Instanz die Möglichkeit einer palliativen Sedierung mit dem vorrangigen Ziel der Symptomkontrolle. Bei einer palliativen Sedierung wird durch Zuhilfenahme von Beruhigungsmitteln ein schlafähnlicher Zustand erzeugt, durch welchen der Patient abgeschirmt wird [6, 16, 30, 38, 48].

Bezüglich der Schmerztherapie wird in der deutschen S3-Leitlinie für Patienten mit einer nichtheilbaren Krebserkrankung Folgendes empfohlen:

Die Schmerztherapie richtet sich nach der Schmerzintensität. Dabei soll versucht werden, ein aufsteigendes Verfahren, ähnlich dem WHO-Stufenschema, umzusetzen. Das WHO-Stufenschema wurde 1986 entwickelt und stellte die erste Form einer Vereinheitlichung in der onkologischen Schmerztherapie dar [49]. Die vier Stufen des WHO-Stufenschemas sind:

1. Stufe: Nichtopioidanalgetika (z. B. Metamizol, Ibuprofen etc.)
2. Stufe: Niedrigpotente Opioide (z. B. Tramadol, Tilidin etc.) + Stufe 1
3. Stufe: Hochpotente Opioide (z. B. Morphin, Fentanyl etc.) + Stufe 1
4. Stufe: Invasive Verfahren (z. B. Nervenblockaden, Neurolysen etc.) [20, 42].

Heutzutage orientiert man sich immer noch an dieser Vorgabe, ist in der Umsetzung aber nicht mehr so stringent wie noch vor wenigen Jahren [5]. So haben z. B. Raffa und Pergolizzi die Schmerzpyramide entwickelt, wodurch die Schmerztherapie dynamischer erscheint als bei dem WHO-Stufenschema [31]. Primär sollen lang wirksame Opioide (z. B. Morphin, Oxycodon, Hydromorphon) oral verabreicht und eine kurz wirksame Komponente, welche schnell über die sublingualen, bukkalen oder nasalen Schleimhäute aufgenommen werden kann, für den akuten Durchbruchschmerz zur Verfügung gestellt werden. Erst bei Versagen der oralen Aufnahme soll auf die transdermale, subkutane oder intravenöse Applikationsform gewechselt werden. Koanalgetika und Nichtopioide werden als Begleitmedikation zur Steigerung der Wirksamkeit empfohlen. Bei dadurch nicht ausreichend behandelbaren Schmerzzuständen kann über alternative Verfahren, z. B. lokale oder spinale Nervenblockaden, nachgedacht werden [20, 42].

## Was gibt es bereits zu dieser Thematik?

In der Literatur fanden sich Anfang der 2000er-Jahre Hinweise auf eine Unterversorgung von Patienten mit Tumorschmerz sowie auf eine mangelnde Dokumentation der Schmerzsymptomatik im ambulanten Bereich der palliativen Versorgung [34, 45]. Durch den Ausbau der ambulanten Palliativversorgung in den letzten 20 Jahren ist anzunehmen, dass die Versorgung schwersterkrankter und sterbender Menschen insgesamt besser geworden ist, doch begleitende Studien hierzu fehlen [23, 29]. Dabei ist bekannt, dass sich Menschen insbesondere vor einem schmerzhaften Tod fürchten und gerade Sterbende sowie ihre An- und Zugehörigen einen friedlichen und schmerzfreien Tod der Patienten wünschen [22]. Iris Borchmeyer beschreibt in ihrer Dissertation (2003) die Verbesserung der Schmerztherapie durch die Aufnahme in einem Hospiz. Sie konnte eine signifikante Senkung der Schmerzintensität nach der numerischen Rating-Skala (NRS) von 6,4 ± 2,87 auf 2,5 ± 2,69 nach der Aufnahme zeigen. Die mangelnde Schmerztherapie vor Aufnahme ins Hospiz konnte sie vor allem auf Dosierungsfehler der Basismedikation und fehlende Begleitmedikamente zurückführen [4]. In Italien konnte Elisabetta Petracci mit ihrer Forschungsgruppe ähnliche Ergebnisse 2016 publizieren. In der untersuchten Kohorte ging der mittlere NRS-Wert von 2,58 ± 2,61 innerhalb von sieben Tagen nach Hospizaufnahme auf 1,40 ± 1,72 (*p* = 0,002) zurück. Besonders signifikant war der Rückgang bei Patienten, die bei Aufnahme ins Hospiz bereits stärkere Schmerzen ≥ 4 hatten. Hier konnte die Schmerzintensität durch die Behandlung von 5,51 ± 1,24 auf 1,76 ± 1,91 gesenkt werden. Sie beobachtete auch eine Zunahme der parenteralen Verabreichung (z. B. intravenös oder subkutan) von Opioiden während des Behandlungszeitraums [27]. Ähnliche Ergebnisse demonstrierten Ehrlich und Walker 2016 in ihrem Review, wobei die Schmerztherapie nach Hospizaufnahme wesentlich bessere Wirkungen zeigte und die Lebensqualität dadurch gesteigert werden konnte [8]. Haakon Sandvold und Kollegen konnten in ihrer retrospektiven Untersuchung darstellen, dass eine Anpassung der Schmerztherapie durch die SAPV bei Patienten, die zuvor vom Hausarzt oder einer Schwerpunktpraxis betreut wurden, wesentlich früher stattfinden musste als bei Patienten, die aus einem onkologischen Zentrum zugewiesen wurden [35]. In der Auswertung des schwedischen Gesundheitsregisters durch Klint und Kollegen 2019 konnte demonstriert werden, dass ein Viertel der Verstorbenen in der letzten Phase ihres Lebens unter nicht ausreichend behandelten Schmerzen litt. Dabei legte die statistische Analyse offen, dass das relative Risiko, unterversorgt zu sein, im Krankenhaus signifikant höher war als in speziellen palliativmedizinischen Einrichtungen [17].

Nach unserem Kenntnisstand gibt es bisher keine Untersuchung, in der die generelle schmerztherapeutische Versorgung von Patienten in der spezialisierten ambulanten Palliativversorgung in Deutschland erhoben wurde. In persönlichen Gesprächen mit Kollegen konnten wir jedoch eine Uneinheitlichkeit in Bezug auf die Handhabung der Schmerztherapie in verschiedenen Sektoren der palliativmedizinischen Versorgung feststellen. Deshalb soll mit dieser Querschnittsstudie erhoben werden, welche Schmerztherapieverfahren aktuell in der ambulanten Versorgung palliativer Patienten durch die SAPV-Teams angewendet werden.

## Methodik

Nach positivem Ethikkommissionsvotum (Aktenzeichen: 67/21; Ethikkommission der Philipps-Universität Marburg, Deutschland; DRKS Reg.-Nr.: 00026132) wurden am 27.05.2021 alle bei der Deutschen Gesellschaft für Palliativmedizin online gelisteten deutschen SAPV-Teams (insgesamt 307) postalisch angeschrieben und um Teilnahme an der Erhebung gebeten [50, 51]. Jedem SAPV-Team wurde ein Fragebogen inklusive Anschreiben sowie ein vorfrankierter Rücksendeumschlag zugesandt. Mit dem Anschreiben wurde eine Person aus der Leitungsebene (Teamleitung, Pflegeleitung, leitender Arzt) gebeten, stellvertretend für das Team an der Erhebung teilzunehmen. Die Daten wurden mithilfe von SPSS (Version: IBM SPSS Statistics 28, IBM Deutschland GmbH, Ehningen, Deutschland) rein deskriptiv ausgewertet. Die vorhandenen Freitextfelder wurden inhaltlich miteinander verglichen und nach gemeinsamen Themen kategorisiert und ausgewertet.

## Ergebnisse

**Abb. 1:**
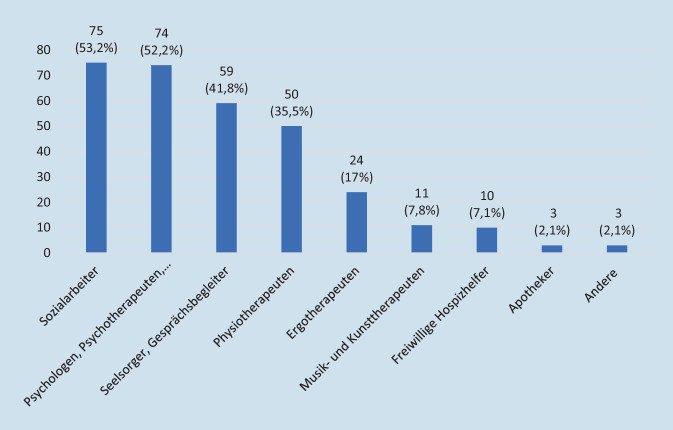
Berufsgruppen der SAPV (*n* = 141), ausgenommen Ärzte und Pflegekräfte

Insgesamt erhielten wir in einem Zeitraum von knapp vier Monaten (27.05.–15.09.2021) 175 (57 %) Fragebögen ausgefüllt zurück. Der Tab. 1 sind die demografischen Daten der antwortenden SAPV-Teams zu entnehmen. Neben Ärzten und Pflegekräften arbeiten in der Patientenbetreuung auch viele weitere Berufsgruppen, vor allem Sozialarbeiter, Psychologen und Physiotherapeuten, zusammen (vgl. Abb. 1).

**Tab. 1:** Demografische Angaben der teilnehmenden SAPV-Teams

	Total	%
*Wer füllt den Fragebogen aus? (Mehrfachantwort möglich)* (*n* = 173)		
SAPV-Leitung	56	32,4
Zuständige(r)/leitende(r) Arzt/Ärztin	93	53,8
Pflegerische Leitung	24	13,9
Andere Berufsgruppe: (z. B. Koordinator/-in)	4	2,3
*Wie viele Mitarbeiter arbeiten in Ihrer SAPV?* (*n* = 172)		
1–10	47	27,3
11–20	81	47,1
21–30	24	14
31–40	5	2,9
> 40	15	8,7
*Wie viele Patienten betreuen Sie durchschnittlich pro Jahr?* (*n* = 172)		
1–100	28	16,3
101–200	18	10,5
201–300	28	16,3
301–400	29	16,9
> 400	69	40,1
*Arbeiten Sie in Kooperation mit einem Hospiz oder Krankenhaus?* *(Mehrfachnennung möglich)* (*n* = 174)		
Nein	22	12,6
Ja, in Kooperation mit einem Hospiz	131	75,3
Ja, in Kooperation mit einem Krankenhaus	124	71,3
*Betreuen Sie Kinder oder erwachsene Patienten?* (*n* = 174)		
Ausschließlich Erwachsene	136	78,2
Ausschließlich Kinder	13	7,5
Sowohl Kinder wie auch Erwachsene	25	14,4

*SAPV* spezialisierte ambulante Palliativversorgung

**Abb. 2:**
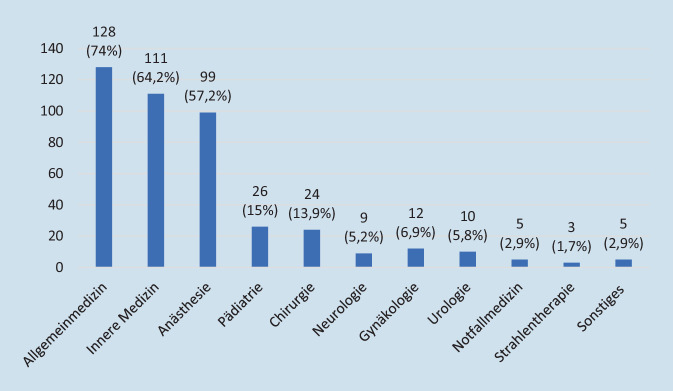
Fachrichtungen der Ärzte in der SAPV (*n* = 173)

Die in der SAPV tätigen Ärzte sind zum größten Anteil Allgemeinmediziner, Internisten, Anästhesisten und Chirurgen. Zu einem untergeordneten Anteil finden sich auch weitere Fachdisziplinen. In pädiatrischen SAPV-Teams sind entsprechend Pädiater angestellt (vgl. Abb. 2). Der überwiegende Anteil der SAPV-Dienste arbeitet in Kooperation mit einem Hospiz (75,3 %) und/oder Krankenhaus (71,3 %). Der Großteil der Teams versorgt ausschließlich erwachsene Patienten (78,2 %) im häuslichen Umfeld, einige Anbieter sowohl erwachsene als auch pädiatrische Patienten (14,4 %) und ein geringer Anteil ausschließlich Kinder und Jugendliche (7,5 %).

Für die Dokumentation von Schmerzen werden überwiegend die numerische Rating-Skala (NRS) (79,9 %), die verbale Rating-Skala (VRS) (40,8 %) sowie die visuelle Analogskala (VAS) (37,9 %) genutzt. Zu einem geringen Anteil werden auch spezielle Schmerzfragebögen (10,9 %) eingesetzt und lediglich vier Teams geben an, Schmerzen nicht zu dokumentieren (2,3 %). Nahezu alle SAPV-Teams (94,8 %) erstellen individuelle Schmerztherapiekonzepte für die Patienten und nur 5,2 % gehen nach standardisierten Therapieschemata vor. Bei der Erstellung der Schmerztherapiekonzepte orientieren sich 88,2 % am WHO-Stufenschema. Darüber hinaus geben 70,9 % der Befragten an, dass sie die S3-Leitlinie der Deutschen Gesellschaft für Palliativmedizin kennen und auch in der täglichen Arbeit umsetzen. 25,6 % der Befragten kennen die Leitlinie zwar, aber setzen diese nicht um, und bei lediglich 3,5 % ist die Leitlinie nicht bekannt.

## Formen der Schmerztherapie

Für die Verabreichung der Schmerzmedikamente nutzen fast alle SAPV-Teams mehrere Möglichkeiten (oral retardiert 98,9 %; oral akut 98,9 %; subkutan 97,7 %; transdermal 96 %; intravenös 89,7 %; bukkal 86,8 %; nasal 82,2 %; sonstige 24,7 %). Es werden auch alle gängigen Schmerzmedikamente eingesetzt (Tab. 2), hier zeigen sich jedoch Unterschiede in der Häufigkeit der Verwendung. Metamizol als Nichtopioidanalgetikum wird bei fast allen Teams genutzt (98,4 %), ebenso wie Morphin (98,3 %), Hydromorphon (97,1 %) und Fentanyl (96 %) aus der Substanzgruppe der Opioide. Bei den Koanalgetika werden vor allem Pregabalin (96,6 %) und Dexamethason (94,9 %) am häufigsten eingesetzt. Ein geringer Anteil gibt explizit an, Cannabis und Derivate (2,9 %) oder Methadon und Derivate (10,9 %) nicht zu verwenden. Für die Prophylaxe gegen Koprostase werden von 93,7 % der Befragten Laxanzien regelhaft verordnet. Für die Behandlung von Durchbruchschmerzen nutzen bis auf ein Team alle Teams eine Form der patientenkontrollierten Schmerztherapie (PCA). Dabei wird von den meisten SAPV-Teams die subkutane (93,1 %) und intravenöse (90,8 %) Applikationsform angeboten (z. B. durch entsprechende Schmerzpumpen oder eingewiesene Angehörige), gefolgt von oralen (43,9 %), sublingualen (39,3 %), nasalen (34,1 %) und rektalen (17,9 %) Verabreichungsmöglichkeiten (z. B. kurz wirksame Rescue-Medikation am Bett). Regionalanästhesieverfahren werden in der häuslichen Umgebung fast gar nicht zur Schmerztherapie genutzt (nur 13,4 %). Einige wenige SAPV-Teams geben jedoch an, peridurale Schmerzkatheter (5,8 %), periphere Schmerzblockaden (2,9 %) oder aber alle Formen der Regionalanästhesie (4,7 %) anzubieten. Auch tumorspezifische Behandlungsverfahren und invasive Schmerztherapien werden im ambulanten Bereich durch den Großteil der SAPV-Teams supportiv eingesetzt (Tab. 3). Von vielen SAPV-Teams (62 %) werden auch alternative Behandlungsoptionen in der Schmerztherapie angeboten. Aroma(öl)therapien, psychologische Unterstützung im Sinne von Gesprächstherapien oder der Entwicklung von Coping-Strategien sowie Physiotherapie, Massagen, Meditation, Hypnose und progressive Muskelrelaxation werden besonders häufig genutzt. Seltener kommen Akupressur, Akupunktur, Atemtherapie, TENS, thermische Verfahren, Wickel und Auflagen sowie Kunst- und Musiktherapie zum Einsatz (Tab. 4).

**Tab. 2:** Eingesetzte Analgetika und Koanalgetika

	Total	%
*Nichtopioidanalgetika* (*n* = 174)		
Metamizol	173	99,4
Ibuprofen	122	70,1
Paracetamol	96	55,2
Coxibe	93	53,5
Diclofenac	64	36,8
ASS	18	10,3
*Opioidanalgetika* (*n* = 174)		
Morphin	171	98,3
Hydromorphon	169	97,1
Fentanyl	167	96
Oxycodon	144	82,8
Buprenorphin	125	71,8
Tilidin	120	69
L‑Methadon	108	62,1
Tapentadol	89	51,2
Piritramid	27	15,5
Sufentanil	13	7,5
Remifentanil	13	7,5
*Koanalgetika* (*n* = 175)		
Pregabalin	169	96,6
Dexamethason	166	94,9
Gabapentin	155	88,6
Butylscopolamin	152	86,9
Trizyklische Antidepressiva	151	86,3
Mirtazapin	145	82,9
Ketamin	99	56,6
Clonidin	66	37,7
β‑Blocker	34	19,4
Dexmedetomidin	19	10,9
Sonstiges (freie Angabe): Cannabis	20	11,4

**Tab. 3:** Tumorspezifische Behandlungs- und invasive Schmerztherapieverfahren

	Total	%
*Nutzen/initiieren Sie tumorspezifische Behandlungs- oder invasive Schmerztherapieverfahren? (Mehrfachnennung möglich)* (*n* = 174)		
Nein	28	16,1
Strahlentherapie	146	83,9
Chemo‑/Immuntherapie	117	67,2
Operative Resektion	72	41,4
Neurolyse	27	15,5
Kryoablation	14	8,1
Sonstiges (freie Angaben, z. B.: intrathekale Schmerzpumpen, Truncus-coeliacus-Blockade, Entlastungspunktionen, Botox, Bisphosphonate, Spinalkatheter)	14	8,1

**Tab. 4:** Alternative Verfahren in der Schmerztherapie

	Total	%
*Welche alternativen Verfahren zur Schmerztherapie werden eingesetzt? (Mehrfachnennung möglich)* (*n* = 168)		
Keine	64	38,1
Aromatherapie	35	20,8
Verhaltenstherapie/psychologische Konzepte (z. B. Coping-Strategien)	22	13,1
Physiotherapie	19	11,3
Progressive Muskelrelaxation	17	10,1
Massage	16	9,5
Meditation (z. B. Yoga, Reiki etc.)	15	8,9
Akupressur	14	8,3
Entspannungsübungen	14	8,3
Hypnose	12	7,1
Wickel/Auflagen	8	4,8
Akupunktur	7	4,2
Wärme‑/Kälteanwendungen	6	3,6
TENS (transkutane elektrische Nervenstimulation)	6	3,6
Atemtherapie	6	3,6
Kunst‑/Musiktherapie	5	3
Taping	4	2,4
Homöopathie	3	1,8
Sonstiges (Traumreisen, basale Stimulation, topische Analgesie, komplementäre Pflege)	4	2,4

## Palliative Sedierung

**Abb. 3:**
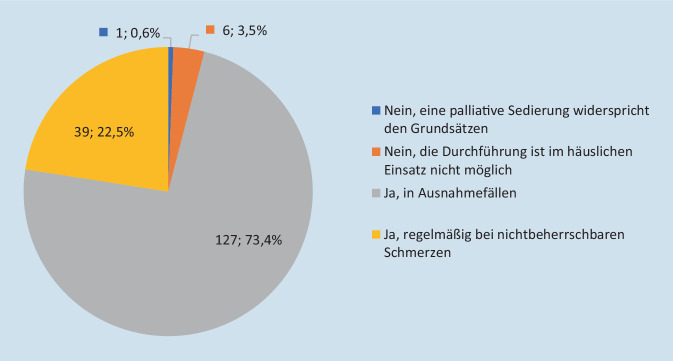
Durchführung einer palliativen Sedierung bei nichtbeherrschbaren Schmerzen (*n* = 173)

**Abb. 4:**
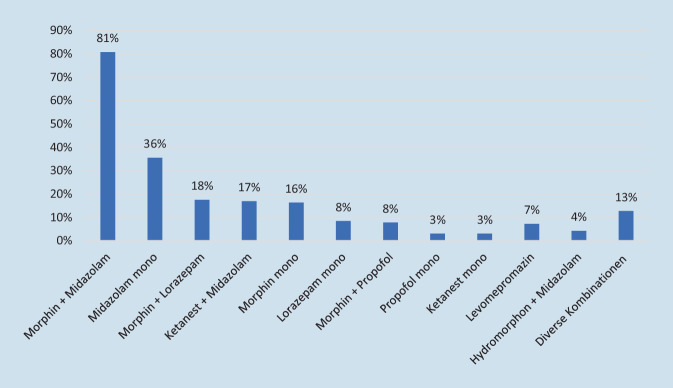
Eingesetzte Medikamente für eine palliative Sedierung (*n* = 166)

Sofern mit der standardmäßigen Schmerztherapie keine ausreichende Symptomkontrolle erreicht werden kann und Patienten unter nichtbeherrschbaren Schmerzen leiden, wenden 22,5 % der Teams regelhaft eine palliative Sedierung an. Bei 73,4 % kann diese maximale Form der Symptomtherapie in Ausnahmefällen erfolgen. Demgegenüber geben sechs Befragte (3,5 %) an, dass eine palliative Sedierung im häuslichen Umfeld nicht durchführbar sei und bei einem Team widerspricht eine palliative Sedierung den eigenen Grundsätzen (Abb. 3). Weitere Indikationen für eine palliative Sedierung sind Dyspnoe (81,6 %), terminale Unruhe (73 %), „total pain“ (62 %), unstillbare Übelkeit und Erbrechen (38,7 %) sowie sonstige belastende Symptome (18,4 %), z. B. plötzliche Tumorblutung, massive Lymphödeme im Kopfbereich oder Status epilepticus. Für eine palliative Sedierung wird am häufigsten die Kombination aus Morphin und Midazolam verwendet (80,7 %), gefolgt von Midazolam mono (35,5 %) und Morphin zusammen mit Lorazepam (17,6 %). Darüber hinaus wird eine Vielzahl von verschiedenen Mischungen aus sedierenden, anxiolytischen und analgetischen Medikamenten verwendet (Abb. 4).

## Diskussion

Die Rücklaufquote von 57 % dieser anonymen institutionellen Befragung kann als hoch angesehen werden und liefert damit einen guten Überblick über die Möglichkeiten der Schmerztherapie in der deutschen ambulanten Palliativversorgung. Trotzdem fällt der Vergleich mit anderen Arbeiten schwer, da die meisten Studien aus dem angloamerikanischen Raum stammen und dort das Versorgungsnetzwerk palliativer Patienten anders aufgebaut ist als in Deutschland. Als „hospice“ sind dort zumeist Institutionen gemeint, die wie die deutsche SAPV eine Versorgung im häuslichen Umfeld gewährleisten, aber auch gleichzeitig Hospize mit teilweise angegliederten Tageshospizen betreiben [15, 40]. Der Großteil der teilnehmenden SAPV-Dienste arbeitet aber auch in Deutschland in Kooperation mit einem Krankenhaus oder Hospiz, sodass hier eine Vernetzung in der Versorgung entsteht. Interessanterweise haben aber 12,6 % der befragten Institutionen angegeben, in keiner Kooperation zu stehen, und das, obwohl gerade die interdisziplinäre Zusammenarbeit im Netzwerk eine der Stärken der Palliativmedizin darstellt. Hier sollte in Erfahrung gebracht werden, welche Gründe dafür vorliegen und wie diese Teams besser eingebunden werden können [46].

In der retrospektiven Auswertung von Anniek Masman und Kollegen konnte festgestellt werden, dass insbesondere Morphin, Midazolam und Haloperidol zu den am häufigsten eingesetzten Medikamenten gerade gegen Ende des Lebens gehören. Der Applikationsweg der Medikamente verändert sich dabei von oral bei Aufnahme der Patienten hin zu intravenös oder subkutan gegen Lebensende. Masman weist in ihrem Artikel darauf hin, dass Therapieregime in der Palliativmedizin meist nur auf Erfahrungswerten aufgebaut sind und es an klaren „guidelines“ mangelt [21]. In Deutschland gibt es mit der S3-Leitlinie „Palliativmedizin für Patienten mit einer nicht heilbaren Krebserkrankung“ eine wissenschaftlich fundierte und praxisorientierte Leitlinie zur Schmerztherapie, jedoch stellen wir in unserer Erhebung fest, dass ein Viertel der Befragten angegeben hat, dass die S3-Leitlinie zwar bekannt ist, aber in der täglichen Arbeit keine Anwendung findet [20]. Gründe hierfür wurden im Fragebogen nicht erhoben, sollten aber in Zukunft evaluiert werden, um die Akzeptanz der Leitlinie zu verbessern. Patric Bialas konnte für die stationäre Versorgung darstellen, dass die Implementierung von Behandlungsstandards zur Schmerztherapie bei Patienten wie Behandlern mit positiven Effekten assoziiert ist [3]. Ob dieser Effekt auch auf die Palliativmedizin übertragbar ist, bleibt vorerst unklar, jedoch geben nur 5,2 % der SAPV-Teams an, nach standardisierten Therapiekonzepten vorzugehen, und der Großteil erstellt individuelle Schmerztherapiekonzepte für die Patienten.

Wie bei Masman festgestellt sind Morphin und Midazolam auch in unserer Erhebung die für die palliative Sedierung am häufigsten eingesetzten Medikamente [21]. Stephanie Stiel und Kollegen haben hingegen erhoben, dass es an einer klaren Definition für palliative Sedierung mangelt und dementsprechend der Vergleich verschiedener Methoden und Institutionen schwerfällt. So unterscheidet das Autorenteam um Stiel zwischen einer leichten und tiefen Sedierung und zeigt auf, dass für eine tiefe Sedierung Lorazepam, Promethazin und (Es‑)Ketamin bevorzugt eingesetzt werden [41]. Die European Association for Palliative Care (EAPC) und auch die Deutsche Gesellschaft für Palliativmedizin (DGP) geben an, dass die palliative Sedierung eine wichtige Therapieoption für Menschen mit hoher Symptomlast ist. Es bedarf hierbei einer hohen fachlichen Expertise der Behandler und enger Kommunikation mit Patienten und Angehörigen über die Maßnahmen und Zielsetzungen. Opioide werden grundsätzlich als ungeeignete Medikamente für eine Sedierung angesehen, sodass auch die Ergebnisse unserer Erhebung nahelegen, dass es weiterhin Unklarheiten in Bezug auf die Definition und die richtige Durchführung von palliativer Sedierung gibt [1, 6, 20]. Trotzdem gilt es im Kontext mit der vorliegenden Erhebung in Bezug auf die Schmerztherapie zu bedenken, dass der supportive Einsatz von Opioiden bei nicht beherrschbaren Schmerzen im Rahmen einer palliativen Sedierung gerechtfertigt sein kann. Auch Anne Hopprich hat in ihrer Auswertung gezeigt, dass 91 % der palliativ sedierten Patienten einer universitären Palliativstation ein Opioid als Komedikation erhalten haben, und dies mit einer notwendigen Symptomkontrolle (Dyspnoe oder Schmerz) gerechtfertigt [13].

Der Großteil der SAPV-Teams hat in unserer Erhebung angegeben, dass sie zur Schmerztherapie auch alternative Behandlungsmethoden einsetzen (vgl. Tab. 4). Insbesondere für die Musiktherapie gibt es einige Untersuchungen, die den Nutzen hierfür belegen können [11, 19, 43], aber auch die Aromaöltherapie bekommt einen immer größeren Stellenwert in der supportiven Behandlung [9, 32]. Diskussionswürdig ist in diesem Zusammenhang jedoch auch, dass 38 % der befragten SAPV-Teams keine solchen alternativen Behandlungsoptionen anbieten. In der Arbeit von Pape et al. konnte am Beispiel von Physio- und Ergotherapie gezeigt werden, dass die Verfügbarkeit im ambulanten Sektor schwierig ist und z. B. durch Fachkräftemangel und aufgrund von Finanzierungsmöglichkeiten erschwert wird [26]. Ob diese Gründe auch die Verfügbarkeit anderer Behandlungsmöglichkeiten erschweren, sollte untersucht werden.

Bei der Betrachtung der eingesetzten Medikamente stellen wir fest, dass eine enorm große Vielzahl an Medikamenten zum Einsatz kommt. Dabei werden auch für die ambulante Versorgung unübliche Medikamente (z. B. Piritramid, Sufentanil oder Remifentanil) angegeben. Die Frage ist, ob ein solch großes Angebot sinnhaft ist oder ob man die Behandlung nicht auch mit einer geringeren Auswahl an Medikamenten bewerkstelligen könnte. Es ist bekannt, dass es gerade bei älteren Patienten oftmals zu einer Polypharmazie (in der Regel die Einnahme von > 5 Medikamenten) kommt. Aber auch bei Palliativpatienten wird dies bis in die letzten Wochen vor dem Versterben beobachtet. Durch die Einnahme mehrerer Medikamente kann es zu Interaktionen und ungewollten Wechselwirkungen kommen, die eine Gefährdung für die Patienten darstellen. In diesem Zusammenhang wird heutzutage der Fokus auf das sogenannte „deprescribing“ gelegt [10, 18, 33, 39]. Untersuchungen haben dabei gezeigt, dass Nebenwirkungen und fehlerhafte Dosierungen seltener vorkommen, wenn die Behandler weniger und dafür ihnen bekannte Präparate verwenden [28, 52]. Dies mag auch damit zusammenhängen, dass nicht jeder Arzt eine umfassende Ausbildung in der Schmerztherapie hat. In diversen Untersuchungen konnte nachgewiesen werden, dass insbesondere im Bereich der ambulanten Versorgung oftmals Schwierigkeiten bei der Tumorschmerzbehandlung existieren [7, 12, 25, 44]. So wird z. B. die Behandlung mit L‑Methadon aufgrund der wechselhaften Pharmakokinetik als schwierig betrachtet und sollte nur von erfahrenen Ärzten angewendet werden [24, 37, 47]. Da es dem Charakter der SAPV entspricht, dass meist mehrere Personen an der Betreuung der Patienten beteiligt sind und somit eine gewisse Kontinuität verloren gehen kann, sollte in den einzelnen SAPV-Teams geprüft werden, ob durch Reduktion des intern zur Verfügung stehenden Medikamentenangebots nicht gleich gute oder gar bessere Therapieerfolge bei geringerer Nebenwirkungsrate erzielt werden können. Vorgegebene Therapieschemata helfen eventuell dabei, in der Schmerztherapie unerfahrene Mitarbeiter zu entlasten. Begleitende Studien zur Reduktion der Angebotspalette an Medikamenten und Etablierung von standardisierten Therapieplänen könnten hierbei die Versorgung im ambulanten Bereich der palliativmedizinischen Versorgung voranbringen.

## Limitationen

Diese Studie ist die erste dieser Art in Deutschland und zeigt, dass die Erforschung der Schmerztherapie im ambulanten Sektor der Palliativversorgung in der deutschen Forschungswelt unterrepräsentiert ist und Vergleiche deshalb hauptsächlich mit angloamerikanischen Studien gezogen werden können, wobei sich die palliativmedizinische Versorgung in diesen Ländern von der deutschen Versorgungsstruktur unterscheidet.

Dem Design des Fragebogens geschuldet hat zumeist immer nur eine Person des SAPV-Teams den Fragebogen ausgefüllt, sodass dadurch systematische Fehler entstanden sein könnten. Insbesondere die Frage nach Kenntnis der S3-Leitlinie ist somit sehr subjektiv und nicht auf alle Mitarbeiter des entsprechenden SAPV-Teams übertragbar. Es wäre insgesamt wünschenswert gewesen, wenn man die gesamte Mitarbeiterschaft hätte befragen können. Dies war aber durch das Studiendesign nicht umsetzbar.

Die Erhebung kann auch keine Aussagen darüber machen, inwieweit die einzelnen Medikamente oder Therapieangebote nun eine besonders gute Schmerztherapie bedingen. Mit der Befragung soll lediglich dargestellt werden, welche Möglichkeiten aktuell zur Verfügung stehen und wie deren Einsatz insgesamt in der Bundesrepublik gehandhabt wird. Dies kann als Ausgangspunkt für weitere Studien dienen.

## Ausblick

Diese Erhebung gibt als erste dieser Art einen Überblick über die Anwendungs- und Durchführungsmöglichkeiten der Schmerztherapie in der ambulanten Versorgung palliativer Patienten in Deutschland. Aus den Daten lässt sich erkennen, dass es eine große Bandbreite von möglichen Angeboten in der Versorgung der Patienten gibt. Schmerztherapie benötigt eine multimodale Behandlung auf verschiedenen Ebenen. Hier zeigt sich, dass unterschiedliche Berufsgruppen in die Versorgung der schwerstkranken Patienten involviert sind und unterschiedliche Strategien, sowohl medikamentös wie auch alternativmedizinisch, angewendet werden. Diese Kombination hat erfahrungsgemäß einen positiven Nutzen, sollte aber durch weitergehende Forschungsarbeit auch evident belegt werden. Ebenso sollte in Erfahrung gebracht werden, ob durch eine Reduktion der angewendeten Medikamente oder einheitliche Therapieschemata positive Effekte erzielt werden können.

## Fazit für die Praxis

- Die Schmerztherapie in der ambulanten Versorgung palliativer Patienten ist heterogen und auf unterschiedliche Arten organisiert.
- SAPV-Teams sind multiprofessionell aufgebaut und arbeiten oftmals in Kooperation mit einem Hospiz und/oder Krankenhaus.
- Eine Kombination aus medikamentösen Behandlungsstrategien mit supportiven und alternativmedizinischen Ansätzen wird überwiegend angeboten.
- Metamizol, Morphin, Hydromorphon, Fentanyl, Pregabalin und Dexamethason sind die am häufigsten verfügbaren Schmerzmedikamente.
- Eine palliative Sedierung kann auch in der häuslichen Umgebung durchgeführt werden, sofern andere Therapien keine Linderung mehr erzeugen.
